# Draft Genome Sequences of Two *Yersinia pseudotuberculosis* ST43 (O:1b) Strains, B-7194 and B-7195

**DOI:** 10.1128/genomeA.00510-13

**Published:** 2013-07-18

**Authors:** Yann Blouin, Mikhail E. Platonov, Christine Pourcel, Vera V. Evseeva, Maxim V. Afanas’ev, Sergey V. Balakhonov, Andrey P. Anisimov, Gilles Vergnaud

**Affiliations:** Université Paris-Sud, Institut de Génétique et Microbiologie, Orsay, Francea; CNRS, Centre National de la Recherche Scientifique, Orsay, Franceb; State Research Center for Applied Microbiology and Biotechnology, Obolensk, Russiac; Irkutsk Antiplague Research Institute of Siberia and the Far East, Irkutsk, Russiad; DGA/MRIS—Mission pour la Recherche et l’Innovation Scientifique, Bagneux, Francee

## Abstract

We report the first draft genome sequences of two *Yersinia pseudotuberculosis* sequence type 43 (ST43) (O:1b) strains, B-7194 and B-7195, isolated in Russia. The total lengths of the assemblies are 4,427,121 bp and 4,608,472 bp, and 3,819 and 4,018 coding sequences, respectively, were predicted within the genomes.

## GENOME ANNOUNCEMENT

To gain insights into the evolutionary origin of the etiologic agent of plague, *Yersinia pestis*, we have analyzed by multilocus variable-number tandem-repeat (VNTR) analysis (MLVA) a collection of 143 Russian isolates of *Yersinia pseudotuberculosis* (1), the species that was shown to be the ancestor of *Y. pestis* (2, 3). Five *Y. pseudotuberculosis* isolates which cluster in the vicinity of *Y. pestis* were further analyzed by multilocus sequence typing (4) and O-genotyping (5) and shown to belong to the globally spread *Y. pseudotuberculosis* clone ST19 O:3. The draft genome sequences of these isolates were recently reported (1). We investigated two strains, B-7194 (alias 58) and B-7195 (alias L-926), which similarly cluster in close vicinity to *Y. pestis*. Multilocus sequence typing (MLST) (4) and O-genotyping (5) data indicate that they belong to ST43 and O:1b, the characteristic sequence type and serotype of the *Y. pseudotuberculosis* lineage sharing the most recent ancestry with *Y. pestis* (3, 4).

Whole-genome sequencing was performed using an Illumina genome analyzer (GA)-IIX (Illumina Inc.) by generating paired-end reads from libraries with an insert size of 300 bp, according to the manufacturer’s instructions. The read length was 100 bp, and the numbers of reads that passed the Illumina quality filters were 11 million for B-7194 and 15.4 million for B-7195, corresponding to 1,100 Mb and 1,540 Mb of high-quality data. For each genome, six million reads were *de novo* assembled using Velvet with a *k* value of 31, and the coding sequences (CDS) were predicted using BioNumerics 7.0 (Applied-Maths, Belgium).

We obtained 240 and 261 contigs larger than 1 kb for B-7194 and B-7195, respectively. The total lengths of contigs were 4,261,235 bp for B-7194 and 4,608,472 bp for B-7195, comprising, respectively, 3,819 and 4,018 predicted CDS. The average G+C content of the draft genome sequences is 47.5%.

### Nucleotide sequence accession numbers.

The draft genome sequences for the strains B-7195 and B-7194 have been included in the European Nucleotide Archive at EMBL-EBI (http://www.ebi.ac.uk/ena/data/view/) under the accession numbers CBKR010000001 to CBKR010000261 and CBKS010000001 to CBKS010000240, respectively.
